# Developing Talent with Artificial Intelligence: Human–AI Symbiotic Theory (HAIST) as a Framework for AI-Mediated Learning and Talent Development

**DOI:** 10.3390/jintelligence14050086

**Published:** 2026-05-19

**Authors:** John C. Chick, Laura Thomsen Morello

**Affiliations:** College of Engineering, Business & Education, University of Bridgeport, Bridgeport, CT 06604, USA; lmorello@bridgeport.edu

**Keywords:** Human–AI Symbiotic Theory, HAIST, talent development, adaptive learning, complementary intelligence, cognitive development, AI in education, non-traditional learners, personalized learning

## Abstract

Traditional talent development models were designed before the AI revolution and do not consider artificial agents as possible sources of development. artificial intelligence is quickly infiltrating education spaces—but our thinking about learning has not caught up with how we can productively pair learners with both human and artificial intelligence. Addressing this gap, we introduce Human–AI Symbiotic Theory (HAIST), a novel theoretical framework designed for AI-facilitated environments, which posits how learners can productively leverage both humans and AI as “development partners” across the entire talent development process. We begin with a comprehensive integration of ideas and theory from the literature on talent development, AI for learning, and human–AI collaboration and use these insights to build HAIST for the specific context of talent development. HAIST comprises three mechanisms—Complementary Intelligence Activation (CIA), Dynamic Adaptive Co-Regulation (DACR), and Agency-Preserving Scaffolding (APS)—that are grounded in prior theory and research on topics like sociocultural theory, self-regulated learning, and distributed cognition. We then demonstrate how HAIST can be applied throughout all phases of talent development while highlighting implications for traditionally underserved learners like adult learners, student veterans, multilingual learners, and first-generation learners. We provide an applied example of how the three mechanisms work in tandem to support talent development and discuss points of tension that must be navigated when applying HAIST (e.g., between adaptation and optimization vs. agency). Lastly, we highlight how considerations of ethics and learner rights (algorithmic bias, learner voice, etc.) should be considered when operationalizing HAIST. Overall, HAIST can serve as a foundational theory to not only understand how talent development should occur between learners and both humans and AI, but also to consider the process of instruction design in AI-mediated learning environments.

## 1. Introduction

Determining what constitutes talent, how to develop it, and how to measure it has been a focus of research within psychology, education, and cognitive science for well over a century. Whether through Binet’s original intelligence tests, Renzulli’s three-ring conception of giftedness, or Gardner’s multiple intelligences framework, theorists have long acknowledged the difficulty of comprehensively defining what it means to be talented (Gardner, 1983; Renzulli, 1978; Sternberg, 1985). The arrival of artificial intelligence (AI) into educational settings has given rise to new iterations of this question: how do we define and understand talent… and how do AI tools contribute to its development?

Recent advances in AI—including but not limited to machine learning algorithms, large language models (LLMs), adaptive learning platforms, and generative AI—have begun to significantly impact educational practice. With applications that include personalized instruction, automated feedback, real-time behavioral assessment, and even creative brainstorming, it is clear that AI has the potential to enhance how we educate students in ways that allow for differentiation at unprecedented scale. But as with any technological solution in education, concerns have been raised about equal access, transparency, perpetuation of bias, and more, for some, the risk that AI will “reduce” learning and creativity to a dataset that can be parsed by machines (Zawacki-Richter et al., 2019; Holmes et al., 2021).

What remains underspecified in discussions about AI in education is how AI might be leveraged to improve our understanding of learning and cultivation of talent in partnership with human intelligence. Classical theories of learning including but not limited to constructivism, connectivism, and self-determination theory were developed largely in the pre-chatbot era and offer an incomplete picture of what human–AI collaboration might look like within educational contexts.

In this paper, we seek to address this gap by presenting Human–AI Symbiotic Theory (HAIST) (Morello & Chick, 2025), a learning framework developed specifically for the age of AI that has strong applications within talent development across cognitive, creative, and socio-emotional domains. Fundamentally, HAIST argues that true human–AI collaboration is realized when human and artificial agents assume complementary (rather than substitutive) roles within a learning partnership, and when the human agent maintains a sense of agency over the direction of that partnership. We propose that human–AI symbiosis of this nature is exactly what talent development requires, allowing us to not automate but amplify the learning process.

A note on the contribution of this paper is warranted. The foundational architecture of HAIST, including its three core mechanisms, was originally introduced in (Morello & Chick, 2025) within a general AI-mediated learning context. The present paper makes a distinct contribution by adapting and extending HAIST specifically to the domain of talent development, a field with its own theoretical traditions, learner populations, and applied challenges. Where the prior publication established HAIST’s theoretical legitimacy through multi-framework validation, this paper applies HAIST to the talent development literature, integrates it with talent identification, nurturing, and assessment frameworks, and develops its equity implications for non-traditional learners in depth. Readers familiar with the prior work will find new theoretical integration, new practical application, and new empirical directions here.

## 2. Literature Review

### 2.1. Theories of Talent Development in Educational Contexts

Talent development has been approached from several perspectives that sometimes overlap but often stay separate from one another. There are psychometric views which focus on the identification of traits, typically cognitive abilities (e.g., Cattell, 1987; Carroll, 1993), developmental views which focus on outside influences (e.g., Bloom, 1985; Gagné, 2004), and humanistic views that focus on motivation, sense of self, and self-actualization (e.g., Maslow, 1943; Deci & Ryan, 1985). Comprehensive talent development models have recently been developed, which take an integrative approach to thinking about how abilities and talent develop, such as Gagné’s Differentiated Model of Giftedness and Talent (DMGT) and Subotnik et al.’s (2011) mega-model of talent development. Both models take stock of how aptitudes, catalysts within the individual, and environmental catalysts contribute to talent development.

A shared criticism of each of these perspectives and models is that they take for granted that talent development occurs within a human-to-human interaction or a human-to-environment relationship. Environmental catalysts are other people such as teachers and mentors, instructional materials, and organizational systems. None of the above scholars conceptualized how an artificial agent could also be a catalyst for one’s development and make significant contributions to identifying, nurturing, and assessing talent, nor has this question been taken up extensively in the literature on talent development.

The lack of theoretical work leaves a void in practice. Artificial intelligence is quickly becoming ubiquitous in learning environments and there is no cohesive set of recommendations for teachers and researchers to draw from about when AI should be used to support talent development, how it should be used, and under what conditions. HAIST aims to fill that void.

### 2.2. AI in Education: Promise and Limitations

The AI in education (AIED) literature has expanded rapidly over the last decade, demonstrating benefits of adaptive learning systems, intelligent tutoring systems (ITS), and more recently generative AI tools like LLMs for supporting learning across contexts (VanLehn, 2011; Luckin et al., 2016; Zawacki-Richter et al., 2019). By leveraging algorithms that respond to learner performance data, adaptive platforms can deliver increasingly personalized content and instruction. ITS are able to deliver hints and feedback tailored to learners. And today, LLMs are showing remarkable ability to respond to learners’ writing with useful commentary and explanation, model ideas with clarity, converse with students using Socratic techniques, and collaborate creatively with learners.

There have also been many examples of limitations in AIEd. Systems built using algorithmic processes often rely on training data from majority-population samples, which can disadvantage learners who do not belong to that group and actually increase opportunity gaps instead of narrowing them (Baker & Hawn, 2022). Questions of transparency are raised by the “black box” nature of many machine learning models, particularly when AIEd is used for purposes of high stakes assessment (Drachsler & Greller, 2016). Concerns have also been raised about the impact of using LLMs for writing development on students’ ability to develop and express their own unique voices, due to the homogeneous fluency of AI-generated text.

Most fundamentally, however, research in AIED has not thoroughly examined how the introduction of AI into learning environments influences the development of students’ underlying intelligence. To date, most research has focused on improvements in performance on near-term learning tasks. But learning scientists and educators are also interested in how AI mediates students’ development of broader capacities like cognitive flexibility, creative thinking, metacognition, and even the socio-emotional skills that correlate with talent development and high achievement. HAIST is proposed as a framework for investigating these issues.

### 2.3. Human–AI Collaboration: Toward a Symbiotic Model

The notion of human–AI symbiosis can be traced theoretically to Licklider’s (1960) original conception of “man-computer symbiosis” whereby human and artificial intelligence work together to “augment” one another. Decades of human–computer interaction research followed suit, investigating how technological tools can augment human thought and action. Distributed cognition theory (Hutchins, 1995) and Vygotsky’s (1978) zone of proximal development have provided important theoretical grounding for much of this work on mediated cognition.

There have also been additional pieces of the literature that have emerged on human–AI teaming concepts, specifically within organizational science and science-of-learning studies. Work within these bodies of research has revealed principles like the notion that proficient human–AI teaming allows both humans and AI to play complementary roles, offloading tasks to the agent (AI or human) with the comparative advantage rather than trying to offload everything the AI can do (Rahwan et al., 2019; Dellermann et al., 2019). Empirical discoveries have been made repeatedly across these studies that when paired with AI, humans perform best when systems are designed to allow them to work together to uplift each other, rather than offloading thinking to the AI. Calls for just this type of symbiotic human–AI teaming have surfaced from the education research literature in recent years as well (Luckin, 2017; Morello & Chick, 2025). But specific theories detailing how this type of symbiosis could be actualized have been few. To that end, it is useful to be clear about what HAIST is and is not, and how it speaks to these adjacent but ultimately distinct frameworks. Hybrid intelligence theory (Dellermann et al., 2019), for example, seeks to explain how human–AI collaboration leads to optimal decision-making performance on organizational tasks, but does not account for how the human collaborator may develop over time or what equity implications may emerge from that partnership. Frameworks around co-regulation (e.g., Zimmerman, 2000; Hadwin et al., 2017) explain how learners might support each other to self-regulate cognition, but were not developed with AI partners in mind and do not describe how AI responsivity should be designed to ensure AI is enhancing human agency rather than replacing it. HAIST borrows the complementarity logic from hybrid intelligence and the developmental scaffolding logic from co-regulation, then grounds both within a nonnegotiable equity mandate for learners who have been traditionally marginalized—a synthesis no one framework was designed to accommodate. As such, HAIST is one attempt at a theory designed specifically for understanding talent development in AI-mediated learning contexts.

### 2.4. Theory-Building Approach

HAIST was constructed through an integrative conceptual synthesis approach, in which the authors conducted a systematic gap analysis across three bodies of the literature: talent development theory, AI in education, and human–AI collaboration research. The process involved identifying shared constructs and structural tensions across these domains, then developing organizing mechanisms capable of bridging the identified gaps. An initial framework was subjected to multi-framework theoretical validation against six established learning and developmental theories, as described by Morello and Chick (2025). The present paper extends that validated architecture into the talent development domain through targeted integration with talent identification, nurturing, and assessment models. This approach is consistent with conceptual model-building in educational research, where theoretical frameworks are developed iteratively through literature synthesis and progressive articulation before empirical testing. It is worth acknowledging that several constructs underpinning HAIST—distributed cognition, adaptive scaffolding, co-regulation—have prior lives in adjacent fields. HAIST’s contribution lies not in inventing these constructs but in reorganizing them around the specific developmental logic of talent cultivation in AI-mediated environments, with an explicit equity mandate for non-traditional learners.

## 3. Human–AI Symbiotic Theory (HAIST): Theoretical Architecture

### 3.1. Core Premises

HAIST is based on four core assumptions that set it apart from previous models of human–machine partnership during learning. Firstly, human intelligence and artificial intelligence should always work together as supplements. While humans are naturally good at reasoning through context, nuance, ethical concerns, creating connections between ideas, understanding meaning, and seeing knowledge relationally, AI lacks these abilities (for now). Artificial intelligence, on the other hand, can process large amounts of information, recognize complex patterns, apply logical frameworks consistently, and create content at scale. Learning happens best when both humans and AI work together intentionally.

Next, true partnership between humans and AI means that both sides adapt to each other. This means that learners need to know how to work with AI intentionally, strategically, and mindfully—we call this AI literacy. Artificial intelligence also needs to responsively adapt to learners and their context, including but not limited to their expertise level, learning objectives, background knowledge, culture, and affect. Static integrations of AI into learning do not align with this idea of symbiosis between learners and technology. Third, learners need to maintain agency. Agency is not just nice to have in talent development situations—it is something that learners need to build through practice if they want to gain meta-cognitive skills, internal motivation, and autonomous learning skills (Deci & Ryan, 1985; Zimmerman, 2000). If an AI system is too optimized for performance, it may take away from the learner’s experience and the true development of talent.

Lastly, HAIST prioritizes equity. Adult learners, student veterans, multilingual learners, first-generation college students, and learners with disabilities are examples of some of the many types of non-traditional learners who have been excluded from traditional models of talent development as well as AI applications. HAIST seeks to incorporate these learners into its framework from the ground up.

### 3.2. The Three Core Mechanisms of HAIST

HAIST offers three primary mechanisms by which human–AI symbiosis facilitates talent development. These mechanisms operate concurrently and synergistically across the learning and development process. They are not discrete phases but continua, along which the talent developer can move to suit the learner’s needs.

Complementary Intelligence Activation (CIA) refers to the moment that learners and AIs determine and deploy their relative intellectual strengths toward learning objectives. In talent development, CIA occurs when the learner utilizes an AI to complete tasks that require extensive data processing (the reviewing literature, gathering data, providing structural feedback), allowing the learner to focus cognitive effort on higher-order tasks (analysis, creation, evaluation, sense-making). This division is critical: CIA is not about outsourcing cognitive work wholesale, which would undermine rather than develop talent. Rather, it is about strategic allocation—the learner must first engage the task deeply enough to identify what is unknown or complex, then deploy AI to address specific processing demands that would otherwise crowd out higher-order thinking. Cognitive load theory supports this design logic: reducing extraneous load through AI assistance can free working memory for the germane cognitive work that drives schema development and genuine domain expertise (Sweller, 1988). CIA thus distinguishes between basic task completion, which AI can handle alone, and talent development, which requires the learner to remain the primary cognitive agent. Ideally, CIA occurs when learners are metacognitively aware enough of their own cognitive processes to understand when to offload work to the AI and when to focus their attention. Metacognitive awareness of this kind is also a skill to be developed—an aspect of cognitive talent that will become increasingly salient in the age of AI.

Dynamic Adaptive Co-Regulation (DACR) refers to the back-and-forth adjustment process by which learners and their AI tools adjust their level of contribution. On the learner’s end of the spectrum, DACR looks like SRL (goal-setting, monitoring, reflection) in response to AI-driven feedback. On the AI end, DACR looks like the system’s responsivity to learner performance, behavioral engagement signals, and requests for assistance. Consistent with equity imperatives central to HAIST, the framework explicitly cautions against reliance on current AI affect-detection technologies, which remain limited in cross-cultural accuracy and risk introducing bias for diverse learner populations (Zeidner et al., 2009). Instead, DACR positions behavioral and performance-based signals—such as response latency, error patterns, and self-reported confidence—as the more reliable and equitable basis for AI responsivity. DACR is critical for talent development because learning at the upper-ends of any domain is rarely linear (Zimmerman, 2000). Recent empirical work underscores the importance of metacognitive support within adaptive AI environments, finding that structured metacognitive scaffolds significantly improve learner self-regulation and learning experience in generative AI contexts (Xu et al., 2025).

**Figure 1:**
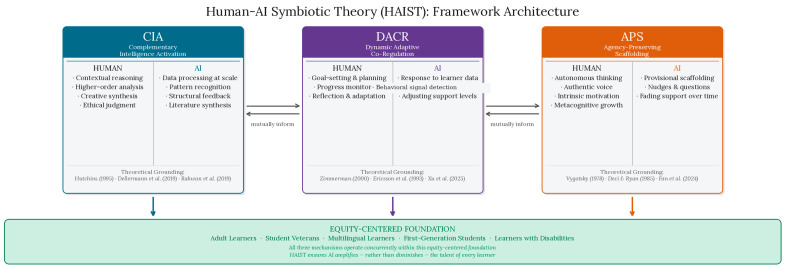
The HAIST Framework: Three interconnected mechanisms of human–AI symbiosis in talent development. Each colored box represents one core mechanism (CIA (Hutchins, 1995; Dellermann et al., 2019; Rahwan et al., 2019), DACR (Zimmerman, 2000; Ericsson et al., 1993; Xu et al., 2025), APS (Vygotsky, 1978; Deci & Ryan, 1985; Fan et al., 2024)), divided into the complementary roles of the human learner and the AI agent. Bidirectional “mutually inform” arrows indicate that each mechanism continuously shapes the others. Downward arrows show that all three mechanisms are embedded within an equity-centered foundation prioritizing non-traditional learners.

Agency-Preserving Scaffolding (APS) refers to the ethical framework within which AI support is provided to the learner. Informed by Vygotsky’s (1978) zone of proximal development, the central tenet of APS is that AI support should be withdrawn as the learner becomes more competent in a given area. In application, this means that AI support should not fully take over a task so that the learner is cognitively disengaged; instead, AI support should nudge, question, and recommend. Applied talent development must preserve the learner’s agency: talent is not developed by simply “asking Google” but through working to solve hard problems. The risk of over-reliance is not theoretical: empirical research has demonstrated that unstructured use of generative AI tools can induce what Fan et al. (2024) call “metacognitive laziness,” a measurable reduction in learners’ self-regulatory engagement and knowledge transfer, even when short-term task performance improves. APS is the HAIST mechanism designed to guard against exactly that dynamic. A question worth addressing directly is how APS fading operates in practice when using generative AI systems like LLMs, which do not automatically reduce their support as learner competence grows. HAIST envisions two complementary pathways. First, educators and instructional designers can build fading into the system design—structuring task sequences so that AI access is progressively constrained as mastery is demonstrated, similar to scaffolded writing curricula. Second, APS can be realized through learner-directed regulation: as AI literacy develops, learners themselves become more strategic about when to invoke AI support, choosing to tackle tasks independently that they previously offloaded. This metacognitive self-regulation is itself a talent to be cultivated, not assumed. Together, the three mechanisms constitute an integrated architecture for talent development, as illustrated in Figure 1 and summarized in Table 1.

#### 3.2.1. Clarifying the Relationship Between CIA and DACR

CIA and DACR are analytically distinct, though they operate in close proximity and can appear to overlap. The clearest way to differentiate them is by their object of focus: CIA governs the allocation of cognitive labor across a learning task at any given moment—which agent does what, and why. DACR governs the regulation of that allocation over time, across a learning trajectory. To use a concrete example: when a doctoral student offloads a literature synthesis task to an AI so they can focus on conceptual argumentation, which is CIA in action. When the same student, over subsequent weeks, finds themselves needing progressively less AI assistance to structure an argument because their own schema has developed, the back-and-forth negotiation underlying that shift is DACR. CIA is the snapshot; DACR is the developmental film. They are not the same process operating at different timescales—CIA describes cognitive role differentiation, while DACR describes the bidirectional regulatory feedback loop through which those roles are negotiated and revised.

#### 3.2.2. Mechanism Tensions and Trade-Offs

A theoretically complete account of HAIST must acknowledge tensions among its mechanisms, not just their synergies. Three tension points deserve explicit attention. First, APS’s commitment to fading AI support can conflict with DACR’s adaptive optimization function, particularly in high-stakes or performance-driven contexts. A system optimizing for near-term performance will tend to provide more support when a learner struggles, which is precisely when APS would call for productive difficulty. HAIST resolves this tension by prioritizing developmental trajectories over task-level performance: the framework instructs that optimization criteria for adaptive AI should be calibrated to long-run competency growth, not short-run task completion. This is a design constraint, not an automatic feature of current adaptive platforms. Second, CIA’s offloading logic can, if poorly implemented, short-circuit the foundational processing that students need to build genuine domain knowledge. Cognitive psychology is clear that some forms of struggle are generative—students who never wrestle with data may not develop the schemas needed to evaluate AI-generated interpretations critically. HAIST addresses this by specifying that offloading should be strategic and metacognitive, not wholesale: learners should engage in the cognitive work until they can identify what they do not know, then use AI to address specific gaps rather than to bypass the task entirely. Third, in many institutional environments, educators have limited control over proprietary AI platform design. HAIST principles cannot always be implemented at the system level. In such contexts, HAIST’s value shifts to the pedagogical layer: instructors can design assignments, assessment criteria, and reflection protocols that instantiate CIA, DACR, and APS principles regardless of what the underlying platform does or does not do automatically.

### 3.3. HAIST and Socio-Emotional Development

A key aspect of HAIST, compared to previous frameworks, is the consideration given to socio-emotional growth as an element of talent development. Socio-emotional skills like self-regulation, perseverance, intellectual tenacity, and teamwork are factors that are frequently found to be present in students who achieve at high levels across both cognitive and creative measures (Subotnik et al., 2011; Zeidner et al., 2009). In educational settings, AI can be used to help develop or harm students’ acquisition of these skills.

According to HAIST, socio-emotional talent development can be enhanced through the use of AI by allowing learners to take creative and intellectual risks in low-stakes environments; modeling good metacognitive habits through the mechanisms of AI-generated feedback; and by automating tasks to allow learners to direct their attention and cognitive resources towards collaborative learning opportunities that AI cannot emulate. On the other hand, HAIST includes dangers to socio-emotional development that need to be addressed, such as learner dependency, intolerance for cognitive struggle, and loss of student voice. Emerging empirical work on human–AI collaborative learning in mixed reality contexts has begun to substantiate these concerns, documenting how the interplay of cognitive and socio-emotional interactions shapes both the quality of collaboration and longer-term learning outcomes (Dang et al., 2025). The talent development implications of these dynamics, particularly in the context of workforce and professional education, have also been taken up in recent scholarship on AI and human resource development (Jung & Yoon, 2025).

## 4. Applications of HAIST in Talent Development Contexts

### 4.1. Talent Identification

Traditional talent identification methods have focused on paper-and-pencil psychometric assessments—IQ tests, standardized achievement measures, aptitude inventories—that represent a narrow slice of human ability and that struggle to account for cultural and linguistic difference (Ford, 2012; Naglieri & Ford, 2005). AI-informed talent identification could broaden the scope of assessment by leveraging behavioral data, process analytics, and natural language processing to pinpoint creative and cognitive aptitudes that might otherwise go unnoticed. At HAIST, AI talent identification follows the principle of Complementary Intelligence Activation: AI detects patterns at scale, and humans provide contextualization, knowledge of the learner’s unique relationship to their learning, and ethical judgment. The partnership creates a sum greater than its parts. On the other hand, HAIST warns against the implementation of AI-only talent identification, which could unintentionally perpetuate current biases at scale and miss outliers whose manifestations of brilliance do not conform to majority-population standards.

**Figure 2:**
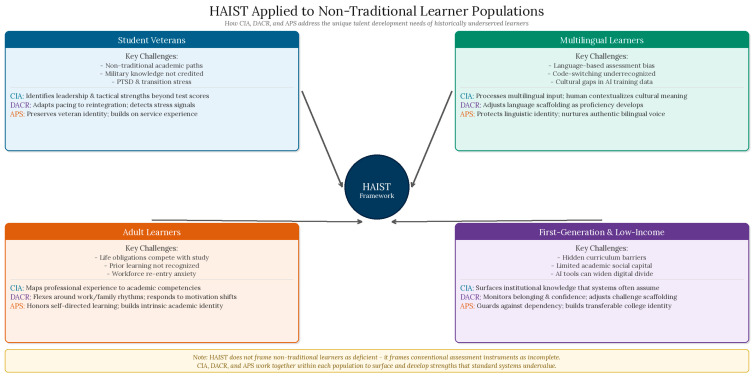
HAIST applied to non-traditional learner populations. The four quadrant panels illustrate how CIA, DACR, and APS each address the specific talent identification, nurturing, and assessment challenges faced by student veterans, multilingual learners, adult learners, and first-generation and low-income students. The central HAIST node represents the framework’s coordinating function across all four populations. The framework positions each population’s challenges as products of incomplete assessment systems rather than individual deficits.

When it comes to supporting the discovery of strengths in non-traditional learners—that is, student veterans who may possess highly tuned cognitive and leadership skills that do not translate into academic test-taking abilities and multilingual learners who may be miscategorized based on assessments rooted in English language fluency—AI talent identification informed by HAIST would work to reveal strengths from a variety of domains and measures, with humans in the loop to provide context. Figure 2 illustrates how each HAIST mechanism maps onto the specific challenges and strengths of these four non-traditional learner populations.

### 4.2. Talent Nurturing

Nurturing talent means providing continuous, differentiated, high-quality learning experiences that are tailored to learners’ developmental pathways. This is exactly where AI’s strengths with respect to scalable personalization can be leveraged—and abused. Real-time feedback and instruction adjusted to learners’ performance through adaptive learning systems allows for the type of deliberate practice known to lead to expert performance (Ericsson et al., 1993). Generative AI can act as a partner for creative thinking, a sounding board for ideas, and a writing instructor to support learners’ cognitive and creative growth across domains.

HAIST considers talent nurturing through the theoretical lenses of Dynamic Adaptive Co-Regulation and Agency-Preserving Scaffolding. Optimizing for how learners perform on near term tasks is not the goal of AI in talent nurturing. Instead, AI should be used to help develop the learner’s own abilities in the long run. This means intentionally fading away support as the learner’s competence grows, allowing them to internalize the skills that were once supported by AI. It also means considering how learners think and feel about their learning journey—making sure AI tools are supporting intrinsic motivation, curiosity, and creative agency. These principles are particularly salient for adult learners, whose unique life-experience assets, prior knowledge, and non-traditional developmental trajectories require AI tools that are genuinely adaptive rather than simply standardized (Adarkwah, 2025). The application of self-determination theory to generative AI contexts further clarifies how AI-mediated learning activities can satisfy learners’ needs for competence, autonomy, and relatedness without becoming a crutch (Chiu, 2024).

#### A Vignette: HAIST in Practice

To ground the framework concretely, consider Marisol, a 42-year-old adult learner returning to higher education after fifteen years in healthcare administration. She is enrolled in a graduate-level program in organizational leadership and has been assigned a research synthesis project. Her instructor has designed the assignment using HAIST principles. Here is how each mechanism operates across the learning episode.

Complementary Intelligence Activation: Marisol begins by identifying her research question—a task her instructor reserves entirely for the human learner, since formulating a meaningful question draws on her lived experience, professional judgment, and intellectual curiosity. Once the question is set, she uses an AI tool to conduct an initial literature scan across fifteen databases, returning 240 abstracts. This is a data-processing task where AI has a clear comparative advantage. Marisol then reads the AI-curated set, makes her own judgments about conceptual relevance and quality, and identifies the three theoretical tensions she will address. CIA is visible here in the deliberate division: AI handles breadth; Marisol handles depth and judgment.

Dynamic Adaptive Co-Regulation: As Marisol drafts her synthesis, she uses an AI writing assistant for feedback on her argument structure. Early drafts generate substantial AI commentary—she is still developing her academic argumentation skills after years away from formal writing. The AI flags logical gaps and suggests transitions. Marisol sets a self-monitoring goal (DACR from the learner side): each week she will attempt one argumentative section without consulting AI before drafting, then compare her unaided draft with AI feedback to identify her growth edges. By week four, AI feedback becomes sparser—not because the system automatically reduces its support, but because Marisol’s writing has improved and her prompts to the AI are now sharper and more targeted. The bidirectional adjustment—her improved self-regulation shaping how she interacts with the AI, and AI feedback shaping her metacognitive awareness—is DACR in action.

Agency-Preserving Scaffolding: The instructor’s course design restricts AI use for the final argumentative synthesis section: Marisol must submit her own unaided draft before receiving any AI feedback. This structural constraint is APS at the course-design level. It ensures that AI does not write the highest-stakes intellectual work for her and that her own analytical voice remains the throughline of the paper. Marisol’s rich professional knowledge—her experience navigating healthcare bureaucracies, managing teams across cultural contexts, and solving adaptive challenges under uncertainty—becomes legible as talent precisely because the assessment requires her to express it. An AI-written synthesis would have rendered that talent invisible. This vignette illustrates how CIA, DACR, and APS are not sequential phases but concurrent design principles that instructors and learners negotiate together across a real learning episode.

### 4.3. Talent Assessment

Assessment is probably one of the more controversial areas of AI application in education because it fundamentally implicates issues of validity, equity, transparency, and constructs that we are attempting to measure. Automated assessment technologies—either in the form of automated essay scoring, behavioral analytics, performance prediction algorithms, etc.—promise speed and consistency while posing new risks to construct validity and fairness (Cope et al., 2011; Madnani & Cahill, 2018).

From the perspective of HAIST, talent assessment is best thought of as a cooperative endeavor: AI brings efficiency and scalability to analysis, formative feedback, and the detection of developmental trends over time. Educators bring contextualized judgment, relationship expertise, and accountability for evaluative decisions that bear significant consequences. Most importantly, HAIST mandates that AI should never be used as the only or ultimate decision-maker in talent assessment—not because AI lacks the capacity for complex, meaningful analysis, but because talent itself is a relational, contextual, and human phenomenon that deserves human judgment in the service of equitable and valid assessment.

In the case of assessments for non-traditional learners, HAIST-informed design would pay particular attention to the likelihood that some AI models may perform suboptimally for learners whose thinking and expression patterns do not match the “norm” of the training data. This includes, but is not limited to, attention to variations in language, background knowledge, and the unique strengths that non-traditional learners develop through life experiences such as military service, adult work and family responsibilities, etc. Table 2 summarizes how HAIST’s three mechanisms apply across all three talent development phases, with explicit attention to equity considerations for non-traditional learners.

## 5. Ethical Considerations

HAIST is designed as an equity-focused framework, and ethical considerations must remain centered throughout the use of HAIST for talent development work. Here we highlight three considerations in particular. First, talent development is vulnerable to the harms of algorithmic bias. An AI system is only as good as the data it is trained on, which means that systems designed to identify and assess talent will inevitably reproduce extant patterns of who counts (and who has counted) as talented. To the extent that systems have historically favored learners from majority culture, language, and class backgrounds, an AI tool is likely to more easily identify talent in learners who reflect characteristics of learners who have previously been identified as talented. In HAIST-based implementation of AI for talent development, practitioners should plan for continual audit and evaluation of AI tools for disparate impact across learner groups, and ensure human judgment is leveraged to catch bias (Floridi et al., 2018).

Second, while unfair or inappropriate responses from AI are a concern in many applications, limiting learner voice is a unique concern raised by the introduction of LLMs. Recent research on student use of AI writing tools expresses concern that students may come to rely on AI-generated language that is fluent but lacks cultural context. But authentic student voice is not simply a nice-to-have in talent development contexts; it is the mechanism by which talent is made visible and impactful. Authentic student voice—whether creative, academic, or intellectual—matters for how students identify as talented and how they are recognized as talented by others. For this reason, HAIST includes the principle of Agency-Preserving Scaffolding, which is intended to protect against AI becoming a crutch for learner thinking and expression.

Third, students deserve to know how the AI tools in their learning environment work. This includes a basic level of transparency about how the tool works and what student data is being collected about them (and how it will be used). Some learners, particularly adult learners and students who do not follow traditional academic paths, may have unique concerns about surveillance, privacy, and how learning data gets leveraged by institutions. When using HAIST to guide implementation, we would prioritize AI systems that make their “thinking” visible and interpretable (Miao et al., 2021).

## 6. Research Agenda and Future Directions

HAIST is offered here as a theoretical starting point meant to be tested, refined, and expanded in light of empirical findings from diverse educational contexts and learner populations. Work along the following lines would represent high-priority areas for future research. The theory’s three core mechanisms—Complementary Intelligence Activation, Dynamic Adaptive Co-Regulation, and Agency-Preserving Scaffolding—should be the focus of empirical work in applied educational settings as soon as is feasible. Research methods that blend behavioral observation, learning analytics, and qualitative interview techniques will be necessary to capture process-level dynamic described by HAIST. Longitudinal mixed-methods studies that trace the development of talent-relevant competencies in AI-augmented learning environments would help validate the framework’s key developmental predictions.

Next, it is vital to create and verify assessment instruments that are consistent with HAIST. To the degree that HAIST’s core mechanisms are both operationally distinct and developmentally meaningful, they should be possible to measure in terms of both presence/intensity and learning outcomes. Work developing such instruments would help validate HAIST empirically while also contributing to the toolkit educators and researchers can draw on when working in AI-augmented talent development contexts. Another important direction involves focusing research efforts on diverse learner groups, such as adult learners and veterans. In light of HAIST’s emphasis on equity, pursuing this line of research seems particularly important for understanding how AI might be leveraged to support, rather than hinder, the talent development of those who have historically fallen through the cracks of traditional education systems.

Research that connects HAIST to ongoing scholarly conversations in cognitive science, psychometrics, creativity research, and human–computer interaction represents a fourth key area for future work. HAIST is intentionally interdisciplinary both in its foundations and its implications; fully realizing the promise of this framework will require input from multiple scholarly communities.

## 7. Conclusions

The emergence of AI as a pervasive educational technology presents both an unprecedented opportunity and an urgent imperative for talent development scholarship. The opportunity lies in AI’s capacity to personalize learning, expand the aperture of talent identification, and provide developmental scaffolding at a scale and quality previously impossible. The imperative lies in the risk that poorly theorized AI integration will reproduce existing inequities, undermine human agency and authentic expression, and reduce the richness of human cognitive and creative development to patterns legible to algorithmic systems.

Human–AI Symbiotic Theory (HAIST) has been introduced as a framework to help meet the imperative and capture the opportunity. HAIST focuses on principles of complementary intelligence, fluid adaptability, and retained human agency to offer theoretical scaffolding and practical direction for designing AI-enhanced talent development systems in ways that enhance human capability. Prioritizing equity and intentionally focusing on the experience of learners who have been historically marginalized or excluded from traditional higher education framings, HAIST’s focus on non-traditional learners such as adult learners, student veterans, multilingual learners, and first-generation students expands the TD conversation beyond the majority-culture learner experiences that have predominated in theory and practice. This does not meant to be an exhaustive list. It is an invitation to continue building.

This article represents an initial theoretical articulation of HAIST in the talent development context. Much empirical work remains. But the framework is grounded in a rich theoretical lineage—from Vygotsky to Licklider, from Renzulli to the current generation of AIED researchers—and its core arguments are consistent with the emerging consensus in human–AI collaboration research that symbiosis, not substitution, is the path toward realizing AI’s potential for human flourishing. We invite scholars, practitioners, and researchers across disciplines to engage critically with HAIST, test its propositions, and contribute to its development.

## Figures and Tables

**Table 1:** Summary of HAIST’s three core mechanisms and their talent development functions.

Mechanism	Core Function	Talent Development Role	Key Theoretical Grounding
**Complementary Intelligence Activation (CIA)**	Distributes cognitive labor between human and AI based on comparative strengths; humans handle higher-order reasoning while AI manages data-intensive processing.	Enables metacognitive development; supports identification and deployment of domain-specific strengths; scales personalized challenge for expert performance.	Distributed cognition (Hutchins, 1995); hybrid intelligence (Dellermann et al., 2019); Rahwan et al. (2019)
**Dynamic Adaptive Co-Regulation (DACR)**	Governs bidirectional adjustment between learner self-regulation and AI responsivity; neither agent is static.	Supports deliberate practice and non-linear development; mirrors the iterative feedback loops essential to expert-level growth.	SRL (Zimmerman, 2000); deliberate practice (Ericsson et al., 1993); Xu et al. (2025)
**Agency-Preserving Scaffolding (APS)**	Ensures AI support is provisional and fading; protects learner agency and intrinsic motivation; guards against dependency and loss of voice.	Positions AI as a developmental scaffold rather than a performance crutch; critical for preserving authentic learner voice and identity.	ZPD (Vygotsky, 1978); SDT (Deci & Ryan, 1985); Fan et al. (2024)

**Table 2:** HAIST applied across the three phases of talent development: key principles, HAIST mechanisms, and equity considerations for non-traditional learners.

Phase	AI Role Under HAIST	Primary Mechanism(s)	Equity Considerations for Non-Traditional Learners
**Talent Identification**	AI detects behavioral, linguistic, and cognitive patterns at scale; humans contextualize findings and apply ethical judgment.	Complementary Intelligence Activation (CIA)	AI-only identification risks perpetuating bias against student veterans, multilingual learners, and adult learners whose strengths are not legible to majority-culture training data (Baker & Hawn, 2022; Adarkwah, 2025).
**Talent Nurturing**	AI delivers adaptive feedback and personalized instruction; fades support as competence grows. See Section 3.2 for full mechanism descriptions.	DACR and APS	Adaptive systems must accommodate life-experience knowledge and non-linear learning trajectories common among adult learners; over-optimization for task performance risks “metacognitive laziness” (Fan et al., 2024) and suppression of learner voice.
**Talent Assessment**	AI provides formative data and trend analysis at scale; human educators retain final evaluative authority.	CIA and APS	Automated assessments must be audited for construct validity across culturally diverse and linguistically varied learner populations; human judgment is non-negotiable when stakes are high (Dang et al., 2025; Floridi et al., 2018).

## Data Availability

The original contributions presented in this study are included in the article. Further inquiries can be directed to the corresponding author.

## Abbreviations

The following abbreviations are used in this manuscript:

HAIST	Human–AI Symbiotic Theory
AI	Artificial Intelligence
AIED	AI in Education
CIA	Complementary Intelligence Activation
DACR	Dynamic Adaptive Co-Regulation
APS	Agency-Preserving Scaffolding
ITS	Intelligent Tutoring Systems
LLM	Large Language Model
UDL	Universal Design for Learning
HSI	Hispanic-Serving Institution
